# Cognitive predictors of longitudinal positive symptom course in clinical high risk for psychosis

**DOI:** 10.1016/j.scog.2021.100210

**Published:** 2021-07-28

**Authors:** Ingvild Aase, Johannes Hendrik Langeveld, Jan Olav Johannessen, Inge Joa, Ingvild Dalen, Wenche ten Velden Hegelstad

**Affiliations:** a TIPS Center for Clinical Research in Psychosis, Clinic for Adult Mental Health Care, Stavanger University Hospital, P.O. 8100, 4068 Stavanger, Norway; b Faculty of Health Sciences, University of Stavanger, 4036 Stavanger, Norway; c Research Department, Stavanger University Hospital, P. O. 8100, 4068 Stavanger, Norway; d Faculty of Social Sciences, University of Stavanger, 4036 Stavanger, Norway

**Keywords:** Clinical high risk for psychosis, Positive symptoms, Cognitive predictors, Verbal fluency, Executive functions, Longitudinal

## Abstract

**Background:**

Clinical High Risk (CHS) for psychosis is a state in which positive symptoms are predominant but do not reach a level of severity that fulfils the criteria for a psychotic episode. The aim of this study has been to investigate whether cognition in subjects with newly detected CHR affects the longitudinal development of positive symptoms.

**Methods:**

Fifty-three CHR individuals fulfilling the criteria for attenuated positive syndrome in the Structural Interview for Prodromal Syndromes (SIPS) were included. At inclusion, all participants completed a neurocognitive battery consisting of tests measuring attention, verbal memory, verbal fluency, executive functions and general intelligence. Cognitive domain z-scores were defined by contrasting with observed scores of a group of matched healthy controls (n = 40). Associations between cognitive performance at inclusion and longitudinal measures of positive symptoms were assessed by using generalised linear models including non-linear effects of time. All regression models were adjusted for age and gender.

**Results:**

Overall, SIPS positive symptoms declined over the time period, with a steeper decline during the first six months. Deficits in executive functions were assossiated witn a higher load of positive symptoms at baseline (p=0.006), but also to a faster improvement (p=0.030), wheras those with poor verbal fluency improved more slowly (p=0.018).

**Conclusion:**

To our knowledge, this is the first study that follows CHR subjects by means of frequent clinical interviews over a sustained period of time. The study provides evidence of an association between executive functions, including verbal fluency, with the evolvement of positive symptoms.

## 1 Introduction

Clinical high risk for psychosis (CHR) is defined as a state in which subthreshold positive psychotic symptoms such as perceptual abnormalities or overvalued ideas occur but are less severe or of too short a duration to fulfil the criteria for a diagnosis of psychosis (McGorry et al., 2003; Yung and McGorry, 1996). CHR has to varying degrees been associated with cognitive deficits (Bora and Murray, 2013; Fuller et al., 2002; Lam et al., 2018). A meta-analysis comparing CHR individuals with healthy controls found impairment in executive functions, general intelligence, verbal and visual memory, verbal fluency, attention, working memory and social cognition (Fusar-Poli et al., 2012), and with those who later converted to psychosis demonstrating more profound deficits in verbal fluency and memory. The literature, however, is inconsistent, with Allott et al. (2019) not finding any such relation. Furthermore, associations between neurocognitive functioning and specific symptom domains in CHR have not been extensively studied. One of the few findings reported relates to poorer neurocognitive performance associated with positive symptoms (Randers et al., 2020). Another study found reaction times for emotion recognition to be negatively associated with positive symptoms (Haining et al., 2020).

The onset of psychosis is marked by positive symptoms such as hallucinations and delusions (Garety et al., 2001) and is associated with deficits in a variety of cognitive functions (Addington et al., 2003; Barder et al., 2013; Bilder et al., 2000; Green, 1996; Rund et al., 2004). Fletcher and Frith (2009) suggest that “*positive symptoms of schizophrenia are caused by an abnormality in the brain's inferencing mechanisms, such that new evidence (including sensations) is not properly integrated, leading to false prediction errors”* (p 56). Along these lines, several researchers (Allen et al., 2012; Hugdahl et al., 2009) posit that auditory hallucinations arise from a cognitive inability to correctly attribute mental events to internal sources, as a result of which they are misinterpreted as arising from external stimuli. These symptoms, as well as thought disorder, arise in the verbal domain; verbal memory and verbal fluency deficits are common in psychosis (Reichenberg et al., 2009; Henry and Crawford, 2005, Henry and Crawford, 2005; Addington et al., 1991; Addington et al., 2016; Becker et al., 2010; Green and Walker, 1985). There is also strong evidence that positive psychotic symptoms are associated with specific executive deficits (Freedman and Brown, 2011; Guillem et al., 2008; Mcgurk et al., 1997; Sabhesan and Parthasarathy, 2005; Williams, 1996) and working memory (Bruder et al., 2011; Gisselgård et al., 2014). Executive functions include inhibition of task-irrelevant responses, working memory, cognitive flexibility (Diamond, 2013; Lehto et al., 2003; Miyake et al., 2000) and verbal fluency (Delis et al., 2001; Henry and Crawford, 2005, Henry and Crawford, 2005). Neurocognitive performance in CHR appears to be at an intermediate level between first episode psychosis (FEP) and healthy controls (Brewer et al., 2006; Eastvold et al., 2007; Hawkins et al., 2004; Kim et al., 2011; Pukrop et al., 2006). Thus, it may be a prelude to the development of positive psychotic symptoms (Addington et al., 2016; Becker et al., 2010; Frommann et al., 2011).

The above findings provide an argument for studying cognitive functioning in conjunction with the development of positive symptomatology along a trajectory from CHR to FEP. Such an approach has the potential to provide a more comprehensive understanding of the co-development of cognitive deficits with positive symptoms. To our knowledge, the present study is the first to address how cognitive functioning in a group of individuals with newly detected CHR may alter the course of further development of positive symptoms longitudinally.

### 1.1 Aims

The aim of this study is to investigate whether performance in cognitive domains in CHR is associated specifically with the longitudinal course of attenuated positive symptoms across a two-year follow-up. We will study deficits in executive functions, verbal memory, verbal fluency, attention and general intelligence shortly after CHR detection and assess their performance as predictors in a statistical model of a longitudinal symptom course.

## 2 Methods

### 2.1 Participants

Fifty-three CHR individuals and forty non-help-seeking healthy controls, matched for age, gender and cultural background, were recruited from the ongoing Prevention of Psychosis (POP) study being conducted at TIPS, a Norwegian early detection of psychosis site (Joa et al., 2015; Joa et al., 2008) located at Stavanger University Hospital. CHR individuals were referred to the study by health-care providers, educators or social service agencies or by self-referral. Healthy controls received compensation of NOK 500 (ca. USD 60). The main inclusion criterion was the fulfilment of criteria for CHR as defined as psychosis-risk syndrome in the Structural Interview for Prodromal Syndromes (SIPS) interview (Miller et al., 2003). Further inclusion and exclusion criteria are described elsewhere (Joa et al., 2021). The healthy controls were recruited locally through social networks (i.e. networks of persons working within our mental health care system). Exclusion criteria included suffering from or being treated for any diagnosable or diagnosed mental disorder, having a first-degree relative with a lifetime history of psychosis, current active substance use or alcohol misuse, neurological disorder or an IQ below 70. For further information regarding the healthy controls, go to Aase et al. (2018).

### 2.2 Clinical measures

We used the Norwegian translated version of the Structural Interview for Prodromal Syndromes (SIPS) (Miller et al., 2003; Miller et al., 1999) version 5.0 (McGlashan et al., 2012) to identify the CHR state. Diagnostic interviews using the Structured Clinical Interview for DSM-IV Axis I Disorders (SCID-I) (First et al., 1994) were conducted by clinical psychologists or psychiatrists.

The SIPS interview assesses positive (five items), negative (six items), disorganised (four items) and general symptoms (four items). The range of the scale for each symptom item is 0–6, where a score of 0 represents the absence of symptoms. Thus, the theoretical maximum scores are 30, 36, 24 and 24 for positive, negative, disorganised and general symptoms respectively. For the positive symptom scale, a score of 6 on any item represents a severe and psychotic state. According to the SIPS interview, there are three different paths to meeting the criteria for a psychosis risk syndrome in correspondence with the definition for the CHR state: (1) Brief Intermittent Psychotic Syndrome (BIPS), (2) Attenuated Positive Symptom Syndrome (APSS) and (3) Lifetime Genetic Risk and Deterioration Syndrome (GRD). APPS is the presence of at least one of the items on the positive symptom subscale at a moderate (=3), moderately severe (=4) or severe but not psychotic (=5) level. All individuals included in the present study fulfilled the criteria for APPS; none were defined as BIPS or GRD.

The modified Global Assessment of Functioning (GAF-M) scale (Hall, 1995) is included in the SIPS interview. GAF-M scores include function and symptom domains. The range of GAF-M is 0–90, where 0 represents the poorest level of function or symptoms.

### 2.3 Procedure

In advance of study inclusion, informed consent was obtained from participants 16 years of age or older. Parents or legal guardians gave informed consent for younger participants. The present study includes all individuals from the overall study (*n* = 99) for whom neuropsychological test results were available (*n* = 53). The inclusion period for neuropsychological testing was four years. Four eligible subjects were not willing to participate during the inclusion period of this part of the POP study.

The SIPS interviews were conducted by extensively trained psychiatric nurses under the supervision of clinical psychologists or psychiatrists. The results of the interview were presented at weekly staff meetings attended by all of the interviewers, supervisors, researchers, psychologists and psychiatrists. The purpose of these meetings was to reach a consensus on the fulfilment of inclusion criteria and diagnoses. The SCID-I reliability for this team is good (*K* = 0.76) (Joa et al., 2007) and the weekly staff discussions minimised the risk of drift.

Clinical assessments took place at study inclusion and were followed up with monthly SIPS interviews for six months, then at nine, twelve, fifteen, eighteen, twenty-one and twenty-four months. The neuropsychological assessment was performed at study inclusion only. CHR individuals received individualised treatment from the secondary mental health services. For more information on treatment characteristics, see Joa et al. (2021).

Thirteen of the fifty-three CHR individuals converted to psychosis during the 24-month period (24.5%). These individuals were offered inclusion in the early detection and intervention in psychosis (TIPS) first-episode study and offered treatment according to national guidelines, including psychopharmacological treatment, continued psychotherapy and multi-family psycho-educational groups.

### 2.4 Neuropsychological assessment and cognitive domains

Clinical psychologists and psychiatric nurses with specialised training administrated neuropsychological testing at baseline.

We used well-recognised tests, including the Delis-Kaplan Executive Function System (D-KEFS) (Delis et al., 2001), the Wechsler Adult Intelligence Scale (WAIS III) (Wechsler, 1997), the Trail Making Test (War Department Adjutant General's Office, W. D, 1944) and the California Verbal Learning Test (CVLT) (Delis, 2000). The dyad of Vocabulary and Block Design (V/BD) (Ryan et al., 1988) is acknowledged as the optimal short form for the assessment of IQ in schizophrenia (Sumiyoshi et al., 2013).

To create a general organisational framework, we grouped the neuropsychological tests into five cognitive functional domains: attention, verbal memory, verbal fluency, executive function and general intelligence. This categorisation was guided by the grouping of tests as presented in a meta-analysis of cognition in first episode schizophrenia (Mesholam-Gately et al., 2009) as well as in a meta-analysis of cognition in CHR individuals (Giuliano et al., 2012).

We chose to compare the CHR subjects with healthy controls rather than established norm groups so as to optimally match controls with CHR subjects. We defined domain scores as the mean z-scores of the included tests contrasted with the observed scores of the healthy controls (*n* = 40). *Z*-scores were computed by subtracting the mean of the scores of the healthy control group and then dividing by the sample standard deviation of the same group.

### 2.5 Statistical analyses

Statistical analyses were performed in IBM SPSS Statistics v. 24 (Spss, 2016) and Stata v. 16. Inspection of boxplots and QQ plots revealed that most of the variables did not follow a normal distribution. Hence, we present descriptive statistics as medians and interquartile ranges (IQR).

The associations between cognitive scores at baseline and longitudinal measures of symptoms scores were assessed using a generalised linear model with a log link (Poisson regression), which is appropriate for use with a moderately skewed outcome variable. Possible overdispersion was handled by using a robust (sandwich) estimation of the standard error (blog.stata.com/2011/08/22/use-poisson-rather-than-regress-tell-a-friend/), retrieved 20.06.21. The functional form of the effect of time was decided, by way of the Akaike and Bayesian information criteria, to be quadratic (candidate models were linear, quadratic, cubic and segmented linear with a break point at six months) in a supplementary analysis including only individuals with at least three valid measurements in models with time effects as the only effects (data not shown). The main analysis included all individuals and all available observations. The models included the fixed effects of baseline cognition, time, time squared, interaction effects cognition by time and cognition by time squared. Correlation between measurements on the same patient was allowed for by including a random intercept in the model. A joint chi-square test of the two interaction effects was used to test if baseline cognition had a statistically significant association with development of symptoms scores over time. Results from regression analyses are presented as unstandardised regression coefficients for the main effects and interaction terms involving cognition, with 95% confidence intervals (CI) and p-values from Wald tests. Since the interpretation of models with both linear and quadratic effects of time is not readily assessable, plots of marginal predicted means over time are presented for the statistically significant results (defined as p < 0.05). All regression models have been adjusted for age and gender and have been performed with the Stata functions “mepoisson”, “margins” and “marginsplot”. A “spaghetti” plot of observed longitudinal developments of SIPS positive symptoms, including a locally weighted regression (“lowess”) curve, was also created in Stata.

## 3 Results

### 3.1 Demographics and clinical characteristics

Table 2 presents the demographics and clinical characteristics of the sample (*n* = 53). Most of the individuals were adolescents between 15 and 19 years of age (median 17, range 13–39). The majority of the individuals were females (58%). 50 of the subjects were born in Scandinavia and three of the subjects were born in other European countries, GAF-M scores were at the moderate to lower part of the scale (median 46, IQR 40 to 55). The individuals had higher scores on SIPS positive symptoms (median of mean item score 2.0) and SIPS negative symptoms (median 1.8) compared with disorganised symptoms (median 0.8). Cognitive domain z-scores at baseline are presented in Table 3.

At baseline, positive symptoms correlated with neither negative (Spearman’'s rho = −0.011, p = 0.94) nor disorganised (rho = 0.099, p = 0.48) symptoms, whereas negative and disorganised symptoms were significantly correlated (rho = 0.503, p < 0.001).

**Fig. 1:**
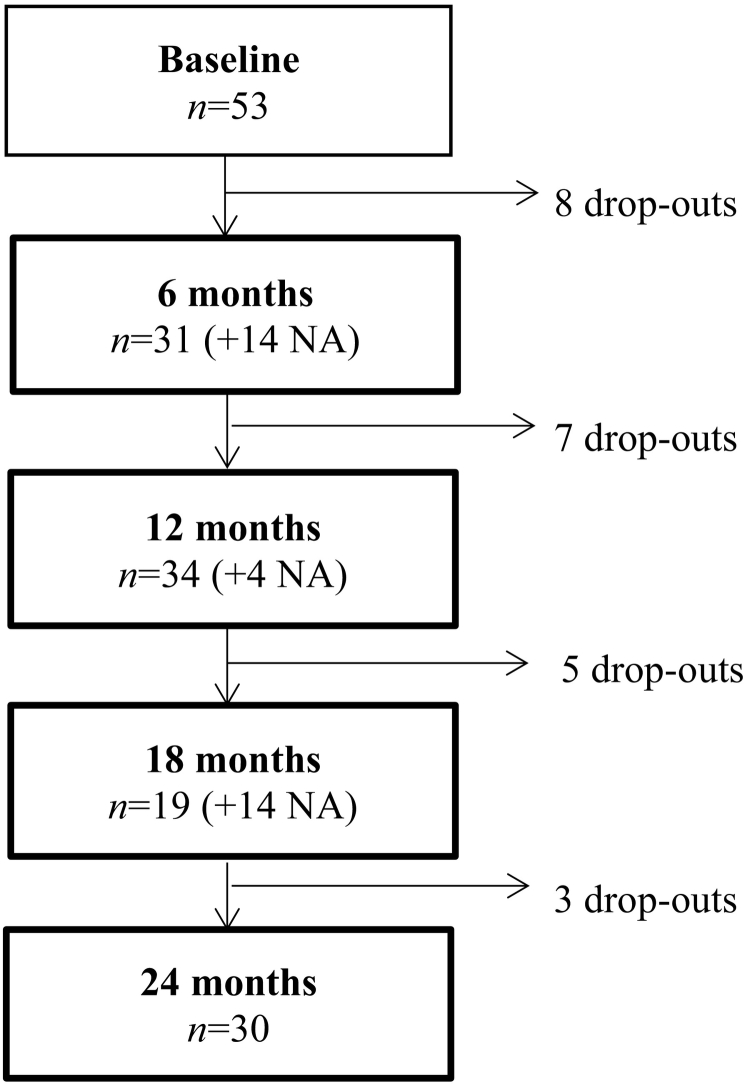
Flow chart of the study cohort Notes: NA = participants not attending assessment at the given time point but continuing to be monitored at later time points.

Figures for individuals dropping out during the 24-month follow up are presented in Fig. 1 (flowchart). Thirty (57%) of the 53 CHR subjects completed the final assessment. Thirteen CHR subjects included at baseline converted to psychosis during the follow-up period. Nine (69%) of these subjects dropped out of the study: four during the first six months, four between six and twelve months, and one after eighteen months.

### 3.2 Development of positive symptom over 24 months

**Fig. 2:**
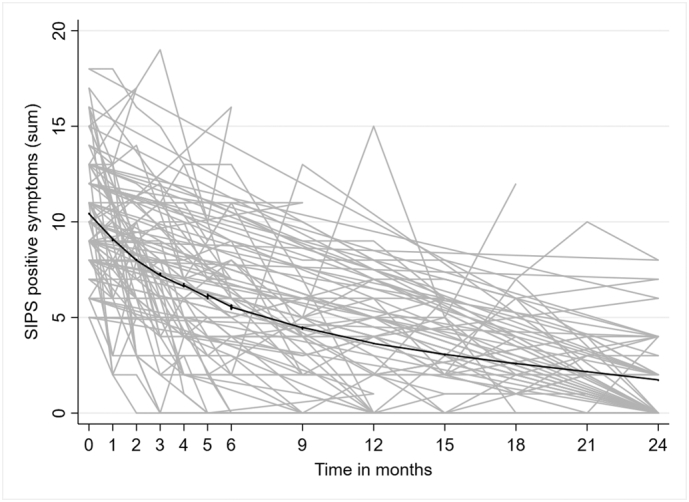
Development of SIPS positive symptoms for CHR subjects over the 24-month follow-up period.

Descriptive statistics for the SIPS positive symptoms scores at the follow-up visits are given in Supplementary Table S1. Fig. 2 illustrates the observed trajectories of positive symptoms over the two-year follow-up. Overall, the symptoms declined over time: sharply during the first six months, followed by a flattening out over the final eighteen months. The mean reduction in positive symptoms from baseline to six months was −4.8 (95% CI, −6.3 to −3.2), *t* = −6.02, p < 0.001 (paired samples *t*-test).

### 3.3 Cognitive functioning and positive symptom course

**Fig. 3:**
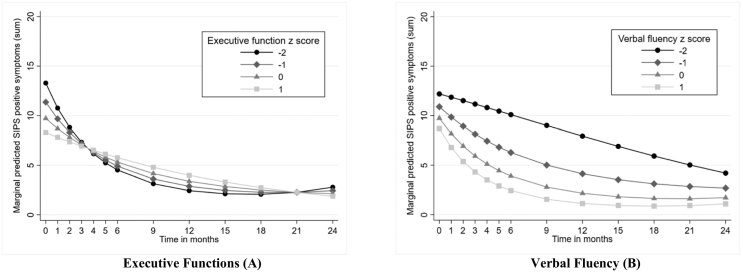
Predicted development of SIPS positive symptoms from cognition at baseline for CHR subjects over a 24-month period Notes: Predicted development of SIPS positive symptoms for 53 CHR subjects over 24 months of follow-up for given values at baseline of (A) executive functions and (B) verbal fluency. Predictions are based on the models presented in Table 4 and Supplementary Table S2. SIPS = Structured Interview for Psychosis-risk Syndromes. CHR = clinical high risk for psychosis.

Performance in the executive domain at baseline was associated with the course of positive symptoms during follow-up (*Χ*^2^ (2 df) = 7.04, p = 0.030 – see Table 4 and Supplementary Table S2). A higher executive domain score was associated with fewer positive symptoms at baseline (p = 0.006) and a slower, more linear decline over the study period as illustrated in Fig. 3A. For a presentation of the individual neuropsychological tests in the executive domain, see Table 1.

**Table 1:** Functional cognitive domains and tests with test variable employed.

Cognitive domain	Name of test	Variable employed
Attention	D-KEFS CWIT Color Naming*	Time to completion (seconds)
	D-KEFS CWIT Word Reading*	Time to completion (seconds)
	WAIS-III Digit Span Forward	No. of correctly reported digits
Verbal memory	CVLT-II List A Total Recall	No. of correct words reported from list A in five trials
	CVLT-II Short-Delay Free Recall: List A	No. of correctly reported words from list A
Verbal fluency	D-KEFS VFT Letter Fluency	No. of correctly reported words (F, A, S)
	D-KEFS VFT Category Fluency (animals)	No. of correctly reported animals
	D-KEFS VFT Category Fluency (names)	No. of correctly reported boys’ names
	D-KEFS VFT Category Switching	No. of correct shifts between categories (fruit, furniture)
Executive functions	WAIS-III Digit Span Backward (***Working Memory***)	No. of correctly reported digits
	D-KEFS CWIT Inhibition* (***Inhibition***)	Time to completion (seconds)
	TMT-B* (Cognitive Flexibility)	Time to completion (seconds)
	D-KEFS CWIT Inhibition/Switching* (***Cognitive Flexibility***)	Time to completion (seconds)
General intelligence	WAIS-III Vocabulary	Accuracy of words defined
	WAIS-III Block Design	No. of correctly produced blocks within time limit

Notes: All variables employed are raw scores. A higher score on the tests indicates a better performance unless tests are marked with an asterix (*), in which a higher score indicates poorer performance. D-KEFS CWIT = Delis-Kaplan Executive Function System Color Word Interference Test (“Stroop”). D-KEFS VFT = Delis-Kaplan Executive Function System Verbal Fluency Test (Delis et al., 2001). WAIS-III = Wechsler Adult Intellegence Scale (Wechsler, 1997). WMS-III = TMT = Trail Making Test (War Department Adjutant General’s Office, W. D, 1944). CVLT-II = California Verbal Learning Test (D. C. Delis, 2000).

**Table 2:** Demographic and clinical data for 53 clinical high risk (CHR) subjects at baseline.

Characteristics	All (*n* = 53)
Age	17 (15-19)
Gender (counts female/male)	31/22
GAF-M at baseline	47 (40-55)
Cultural background (counts Nordic/other European)	50/3
SIPS positive symptoms at baseline	
Sum scores	10 (8-13)
Mean scores	2.0 (1.6-2.6)
SIPS disorganisation symptoms at baseline	
Sum scores	3 (1-4)
Mean scores	0.8 (0.3-1.0)
SIPS negative symptoms at baseline	
Sum scores	11 (6-17)
Mean scores	1.8 (1.0-2.8)

Notes: All data is presented as median (interquartile range) unless otherwise stated. GAF M = Global Assessment of Functioning. SIPS = Structured Interview for Psychosis-risk Syndromes.

**Table 3:** Overview of cognitive domain z-scores at baseline for the 53 CHR subjects.

Cognitive domain	n	Median (IQR)	Min, max
Attention	50	−0.40 (−1.28, 0.19)	−2.43, 1.27
Verbal memory	51	−0.26 (−1.24, 0.48)	−2.76, 1.76
Verbal fluency	52	−0.44 (−0.98, −0.05)	−2.26, 1.50
Executive function	51	−0.21 (−1.05, 0.26)	−2.24, 1.22
General intelligence	53	−0.46 (−1.15, 0.21)	−2.59, 1.49

Notes: Cognitive domain z-scores were defined as the mean z-score of the included tests. IQR = Interquartile range.

**Table 4:** Effect of baseline cognitive domain z-scores on the development of SIPS positive symptoms over 24 months for the CHR subjects (*n* = 53).

		Main effect cognition		Cognition × time		Cognition × time^2^		
Cognitive domain	n/obs	*β* (95% CI)	p	*β* (95% CI)	p	*β* (95% CI)	p	Overall p
Attention	50/371	−0.03 (−0.14, 0.09)	0.66	0.019 (−0.028, 0.066)	0.44	−0.0006 (−0.0025, 0.0012)	0.51	0.73
Verbal memory	51/377	−0.08 (−0.16, 0.01)	0.098	−0.020 (−0.064, 0.023)	0.36	0.0005 (−0.0014, 0.0024)	0.59	0.19
Verbal fluency	52/385	−0.11 (−0.29, 0.07)	0.22	−0.076 (−0.140, −0.012)	0.020	0.0026 (−0.0003, 0.0055)	0.083	0.018
Executive function	51/382	−0.16 (−0.27, −0.04)	0.006	0.052 (0.014, 0.091)	0.008	−0.0021 (−0.0039, −0.0003)	0.020	0.030
General Intelligence	53/396	0.01 (−0.12, 0.14)	0.88	−0.019 (−0.068, 0.030)	0.44	0.0011 (−0.0007, 0.0028)	0.23	0.30

Notes: Results from multilevel Poisson regression models including linear and quadratic effects of time and main effect of cognitive domain z-score, as well as interactions between cognitive score and both time terms. Adjustment has been made for age and sex. Standard errors are robust (sandwich) estimates. P-values from Wald tests. Overall p is for a joint chi-square test of the two interaction terms. SIPS = Structured Interview for Psychosis-risk Syndromes, n = number of subjects, obs = number of observations, CI = confidence interval. See Supplementary Table S2 for complete reports from the models.

Baseline verbal fluency was also associated with the development of positive symptoms (*Χ*^2^ (2 df) = 8.09, p = 0.018). Better verbal fluency was associated with a steeper decline in the first period of follow-up, but with a greater tendency to flatten out, which is illustrated in Fig. 3B. The individual neuropsychological tests in the verbal fluency domain are presented in Table 1.

Performance in the attention domain at baseline was not associated with the course of positive symptoms during the 24-month period (p = 073), nor was performance in the domains of verbal memory (p = 0.19) or general intelligence (p = 0.30).

## 4 Discussion

The present study has investigated whether neurocognition at baseline in CHR individuals is associated with the development of positive symptoms over a 24-month follow-up period.

The main findings are that deficits in executive functions (mental flexibility, inhibition, set shifting) were associated with a higher load of positive symptoms at baseline but also with more rapid improvement, whereas poor verbal fluency was found to be associated with a less favourable development of positive symptoms over the 24-month period. Deficits in attention, general intelligence and verbal memory at baseline were not found to be significantly associated with positive symptoms over time. In general, there was a significant decrease in symptom levels between baseline and the six-month follow-up.

Deficits in language was demonstrated to be a predictor of psychosis development from a longitudinal perspective in a large longitudinal cohort study (*n* = 10,717), and a relative decline in verbal abilities between 13 and 18 years of age was associated with a greatly increased risk for developing schizophrenia (MacCabe et al., 2013). In the present study, we have found that an overall verbal fluency deficit predicts the course of positive symptoms. This is in line with growing evidence in support of deficits in verbal fluency being present in CHR (Fusar-Poli et al., 2012; Giuliano et al., 2012). These deficits are often present during the early stages of positive symptoms and it is suggested that they are a possible predictor of transition to psychosis (Addington et al., 2016; Becker et al., 2010). They are very familiar in psychotic states, particularly in association with positive and negative symptoms (Galaverna et al., 2014). Indeed, verbal fluency has been viewed by some as part of executive functions (Henry and Crawford, 2005, Henry and Crawford, 2005). Thus verbal fluency problems and, in other studies, problems with multiple verbal tasks (verbal memory) may well be regarded as essentially executive problems. Even auditory hallucinations, which are considered to be a misattribution of internal mental events (thoughts, memories), can be thought of as a consequence of executive problems – i.e. difficulties in controlling mental operations. Furthermore, delusions may result from the cognitive dissonance that arises when intrusive thoughts interfere with, or differ from, an individual's established beliefs (e.g. delusions) (Morrison et al., 1995). It follows from these cognitive theories of positive symptoms that delusions are related to inhibition and hallucinations are associated with interference sensitivity, or an inability to ignore irrelevant information (Guillem et al., 2008). Frith (1979) outlined a model for positive symptoms in schizophrenia which proposed that positive symptoms developed due to a failure of the inhibitory process which normally limits the content of consciousness. This model was later supported in schizotypal individuals by Peters et al. (1994). If inhibition skills are impaired, an individual experiencing the CHR state may be more prone to developing perceptual abnormalities and hallucinations due to deficits in inhibiting responses to both internal and external stimuli. In a dichotic listening study at our site (Aase et al., 2018), individuals with CHR were substantially impaired in terms of inhibiting the most salient auditory stimuli when instructed to do so in a dichotic listening paradigm. In the present study, the most salient stimuli for the CHR individuals may be perceptual abnormalities/hallucinations and thus the individuals with better inhibitory skills may be able to ignore, or inhibit, these perceptual abnormalities/hallucinations. Hence, executive functions such as inhibition may serve to protect against further psychosis development. Impairment in mental flexibility is also associated with positive symptoms (Guillem et al., 2008). Deficits in mental flexibility are associated with set shifting and perseverations (Lezak, 2004). This may imply that CHR individuals with poorer mental flexibility have more rigid thinking, which may contribute not only to the maintenance and development of delusions but also to problems shifting sets on the verbal fluency task (e.g. shifting semantic categories and sticking to the right one and shifting letters).

One might further argue that negative and disorganised symptoms can mediate the association found between positive symptoms and cognitive functioning. However, since positive symptoms correlated with neither negative nor disorganised symptoms at baseline, we find it rather unlikely that these other symptoms will play a large role in mediating the relation between cognition and positive symptoms.

All in all, a positive symptom course in CHR appears to be related to executive problems as expressed by verbal fluency and impaired cognitive flexibility.

### 4.1 Strengths and limitations

A major strength of the present study is our frequent symptom monitoring by way of the SIPS interview at 13 time points over a two-year period. To our knowledge, no other CHR study has followed the development of positive symptoms for such a long period after neurocognitive assessment at baseline. Hence, this study provides new knowledge about how executive functions and verbal fluency at baseline may alter the course of positive symptom development over a period of two years.

In respect of age, gender, SIPS symptom levels, global functioning and conversion to psychosis during the study period, our study is comparable to other international studies on CHR samples (Fusar-Poli et al., 2020). This supports the representativeness of our sample and thus the generalisability of its findings. The chosen longitudinal analyses limit the negative effects of individuals who left the study due to conversion or drop-out during a series of observations.

As our sample size was rather small (*n* = 53), firm conclusions cannot be justified.

## 5 Conclusion

Our study is in line with previous studies that provide evidence of executive problems and verbal fluency potentially existing prior to the onset of psychosis. Our findings may be of clinical value in detecting CHR individuals with a higher risk for developing psychosis. More importantly, however, as we found a group effect across all participants regardless of their later conversion, our results provide an argument for viewing the psychological phenomena involved in CHR and FEP as points on a continuum from mental health to mental ill health, including psychosis.

## Ethics

The study was approved by the local Institutional Review Board Regional Committee for Medical Research Ethics Sør-Øst (ref. no. 2009/949). Parents or legal guardians gave informed consent for individuals younger than 16 years of age, as in Norway individuals are legally able to consent without parental approval from the age of 16. The individuals were offered treatment in clinical mental health services at Stavanger University Hospital during the 24-month follow-up period. The present study was conducted according to the requirements of the Declaration of Helsinki/Code of Ethics of the World Medical Association (Association, 2013).

## Funding

The present study received support by way of a personal grant from Health West Foundation to IA (13968), two grants from Health West Foundation (911508 and, later on, 911881). It was also supported by way of a grant (913184) from the Norwegian Extra Foundation for Health and Rehabilitation through EXTRA funds.

## CRediT authorship contribution statement

IA and JHL wrote the first drafts of the paper. IA, ID and JHL conducted the statistical analysis. IA, JHL and WtVH interpreted the results and wrote the second draft of the paper. JOJ and IJ outlined the overall study, Primary Prevention of Psychosis (POP). All of the authors provided detailed comments on the paper over the course of several drafts and contributed to the editing of the final manuscript; they were also available to provide input throughout the process.

## Declaration of competing interest

The authors report no conflict of interest.

## Appendix A Supplementary data

Supplementary data for development of positive symptoms (S1) and regression models (S2). Image 1
