# Supplementary material for: Cognitive predictors of longitudinal positive symptom course in clinical high risk for psychosis
Source: Schizophr Res Cogn. 2021 Jul 28;26:100210. doi: 10.1016/j.scog.2021.100210 (PMC8340303; doi:10.1016/j.scog.2021.100210)
Supplement: Supplementary file 1 — Supplementary data for development of positive symptoms (S1) and regression models (S2). [file mmc1.docx]

**Supplementary Table S1**

Descriptive statistics of sum of SIPS positive symptoms for the included subjects at different assessment time points from baseline to 24 months follow-up

| Time (months) | n | mean (SD) | median (IQR) | min, max | skewness, kurtosis |
| --- | --- | --- | --- | --- | --- |
| 0 | 53 | 10.6 (3.3) | 10 (8, 13) | 5, 18 | 0.304, -0.505 |
| 1 | 33* | 8.7 (3.7) | 9 (6, 11) | 2, 18 | -0.082, 0.092 |
| 2 | 34 | 8.1 (4.1) | 8 (5, 10.25) | 0, 17 | 0.387, 0.096 |
| 3 | 33 | 6.3 (4.6) | 5 (3, 10.5) | 0, 19 | 0.712, 0.372 |
| 4 | 30 | 5.0 (3.4) | 4 (2, 7) | 0, 13 | 0.902, 0.557 |
| 5 | 25 | 4.9 (3.9) | 4 (2, 8) | 0, 13 | 0.571, -0.722 |
| 6 | 31 | 5.9 (3.8) | 5 (4, 8) | 0, 16 | 0.654, 0.365 |
| 9 | 30 | 3.9 (3.6) | 3.5 (0.75, 5.25) | 0, 13 | 0.910, 0.236 |
| 12 | 34 | 3.3 (3.5) | 2 (0, 5) | 0, 15 | 1.365, 2.507 |
| 15 | 23 | 2.9 (2.7) | 2 (0, 6) | 0, 8 | 0.427, -1.396 |
| 18 | 19 | 3.1 (3.3) | 2 (0, 6) | 0, 12 | 1.157, 1.102 |
| 21 | 21 | 2.0 (2.3) | 2 (0, 3) | 0, 10 | 2.081, 6.338 |
| 24 | 30 | 1.7 (2.3) | 0 (0, 3.25) | 0, 8 | 1.333, 0.947 |

*For one subject, one SIPS positive symptoms item was mean imputed

**Supplementary Table S2**

Details of the regression models partly reported in main Table 4.

|  | β (95% CI) | z | p | Χ^2^ (df=2) | Overall p |
| --- | --- | --- | --- | --- | --- |
| **Attention (n=50, obs=371)** |  |  |  |  |  |
| Cogn z score | -0.03 (-0.14, 0.09) | -0.44 | .66 |  |  |
| Time (in months) | -0.12 (-0.17, -0.08) | -5.07 | <.001 |  |  |
| Time^2^ | 0.0027 (0.0009, 0.0045) | 3.07 | .002 |  |  |
| Gender | 0.11 (-0.20, 0.41) | 0.67 | .50 |  |  |
| Age (in years) | -0.002 (-0.035, 0.031) | -0.11 | .91 |  |  |
| Cogn z score x Time | 0.019 (-0.028, 0.066) | 0.78 | .44 | 0.63 | .73 |
| Cogn z score x Time^2^ | -0.0006 (-0.0025, 0.0012) | -0.66 | .51 |  |  |
|  |  |  |  |  |  |
| **Verbal memory (n=51, obs=377)** |  |  |  |  |  |
| Cogn z score | -0.08 (-0.16, 0.01) | -1.65 | .098 |  |  |
| Time (in months) | -0.15 (-0.20, -0.11) | -6.66 | <.001 |  |  |
| Time^2^ | 0.0033 (0.0013, 0.0053) | 3.36 | .001 |  |  |
| Gender | 0.11 (-0.16, 0.37) | 0.78 | .44 |  |  |
| Age (in years) | -0.009 (-0.036, 0.018) | -0.66 | .51 |  |  |
| Cogn z score x Time | -0.020 (-0.063, 0.023) | -0.92 | .36 | 3.32 | .19 |
| Cogn z score x Time^2^ | 0.0005 (-0.0013, 0.0024) | 0.54 | .59 |  |  |
|  |  |  |  |  |  |
| **Verbal fluency (n=52, obs=385)** |  |  |  |  |  |
| Cogn z score | -0.11 (-0.29, 0.07) | -1.23 | .22 |  |  |
| Time (in months) | -0.18 (-0.24, -0.12) | -5.70 | <.001 |  |  |
| Time^2^ | 0.0044 (0.0021, 0.0067) | 3.76 | <.001 |  |  |
| Gender | 0.21 (-0.08, 0.49) | 1.44 | .15 |  |  |
| Age (in years) | 0.010 (-0.017, 0.036) | 0.71 | .48 |  |  |
| Cogn z score x Time | -0.08 (-0.14, -0.01) | -2.32 | .020 | 8.09 | .018 |
| Cogn z score x Time^2^ | 0.0026 (-0.0003, 0.0055) | 1.74 | .083 |  |  |
|  |  |  |  |  |  |
| **Executive function (n=51, obs=382)** |  |  |  |  |  |
| Cogn z score | -0.16 (-0.27, -0.05) | -2.75 | .006 |  |  |
| Time (in months) | -0.11 (-0.16, -0.07) | -4.97 | <.001 |  |  |
| Time^2^ | 0.0021 (0.0001, 0.0040) | 2.10 | .036 |  |  |
| Gender | 0.10 (-0.20, 0.40) | 0.64 | .52 |  |  |
| Age (in years) | -0.004 (-0.037, 0.029) | -0.24 | .81 |  |  |
| Cogn z score x Time | 0.052 (0.014, 0.091) | 2.64 | .008 | 7.04 | .030 |
| Cogn z score x Time^2^ | -0.0021 (-0.0039, -0.0003) | -2.33 | .020 |  |  |
|  |  |  |  |  |  |
| **General intelligence (n=53, obs=396)** |  |  |  |  |  |
| Cogn z score | 0.01 (-0.12, 0.14) | 0.15 | .88 |  |  |
| Time (in months) | -0.14 (-0.20, -0.09) | -5.14 | <.001 |  |  |
| Time^2^ | 0.0034 (0.0015, 0.0054) | 3.44 | .001 |  |  |
| Gender | 0.09 (-0.19, 0.37) | 0.62 | .53 |  |  |
| Age (in years) | -0.002 (-0.035, 0.031) | -0.10 | .92 |  |  |
| Cogn z score x Time | -0.019 (-0.068, 0.030) | -0.78 | .44 | 2.38 | .30 |
| Cogn z score x Time^2^ | 0.0011 (-0.0007, 0.0028) | 1.19 | .23 |  |  |
